# New strategies to advance pre/diabetes care: integrative approach by PPPM

**DOI:** 10.1186/1878-5085-5-S1-A14

**Published:** 2014-02-11

**Authors:** Mahmood S Mozaffari

**Affiliations:** 1 Department of Oral Biology/Pharmacology & Toxicology, College of Dental Medicine, Georgia Regents University, Augusta, Georgia, USA

## 

Predictive, Preventive and Personalized Medicine (PPPM) is the concept of an integrative medical approach based on a paradigm shift from reactive to preventive medicine. The rationale for PPPM relates to the recognition that a) prediction of populations at risk for a disease (e.g., diabetes mellitus) should lead to prevention of its development and progression and b) treatment/intervention strategies should be on the basis of individualized patient information profile. This integrated approach should provide the opportunity for a better approach to health promotion thereby reducing morbidity and mortality and associated costs.

One of the most pressing health-related challenges is the worldwide prevalence of diabetes mellitus, particularly type 2, and associated complications. Due consideration of this reality has led the European Association for Predictive, Preventive and Personalized Medicine (EPMA) to consider Diabetes Mellitus as one of its focal points of activities as exemplified by the introduction of the book entitled: *“New Strategies to Advance Pre/Diabetes Care: Integrative Approach by PPPM”* (Springer 2013). It provides the readership with excellent reviews reporting emerging information regarding global burden of the disease, its associated complications (e.g., retinopathy, nephropathy and impaired wound healing), pathogenic mechanisms, predictive and prognostic biomarkers as well as emerging preventive strategies and therapies, among others. This book is a timely and helpful addition to the growing list of scientific contributions in the field.

